# A Formal Validation Approach for XACML 3.0 Access Control Policy

**DOI:** 10.3390/s22082984

**Published:** 2022-04-13

**Authors:** Carmine Caserio, Francesca Lonetti, Eda Marchetti

**Affiliations:** 1 Computer Science Department, University of Pisa, 56127 Pisa, Italy; carmcase2@gmail.com; 2 ISTI-CNR, 56124 Pisa, Italy; eda.marchetti@isti.cnr.it

**Keywords:** XACML 3.0 formalization, coverage criteria, policy testing

## Abstract

Access control systems represent a security mechanism to regulate the access to system resources, and XACML is the standard language for specifying, storing and deploying access control policies. The verbosity and complexity of XACML syntax as well as the natural language semantics provided by the standard make the verification and testing of these policies difficult and error-prone. In the literature, analysis techniques and access control languages formalizations are provided for verifiability and testability purposes. This paper provides three contributions: it provides a comprehensive formal specification of XACML 3.0 policy elements; it leverages the existing policy coverage criteria to be suitable for XACML 3.0; and it introduces a new set of coverage criteria to better focus the testing activities on the peculiarities of XACML 3.0. The application of the proposed coverage criteria to a policy example is described, and hints for future research directions are discussed.

## 1. Introduction

Security is a challenging issue in modern networked systems where a huge amount of data are managed and exchanged in everyday life. In particular, the privacy and confidentiality attributes of personal and critical data require adequate security mechanisms to be put in place [1]. In this context, access control systems represent an important component for the overall security because they are able to mediate all requests of access. This ensures the protection of data and assures that only the intended, i.e., authorized users can access them. In particular, specific rules can be defined for establishing under which conditions a subject’s access request to a resource can be permitted or denied.

In this context, attribute-based access control (ABAC) [2] systems are the adopted means for enhancing a fine-grained access control. ABAC relies on the combination of various attributes of authorization elements into access control decisions. In the literature, there are several languages for specifying access control policies [3], and among them, the OASIS eXtensible Access Control Markup Language (XACML) [4] is the most commonly used standard in many real-world systems for defining ABAC policies in the XML-based syntax. The XACML language is also widely used to guarantee access control decisions for the distributed Internet of Things (IoT) environments [5,6].

The management of real access control policies is in practice difficult and error-prone [7] due to the verbosity and complexity of the XACML syntax. Faults in the access control policies are very critical because they could open the path to security flaws: either denying accesses that should be authorized or allowing accesses to non-authorized users.

Thus, verification and validation become key issues for XACML policies specification and their implementation [8].

However, in the literature, the commonly available test cases’ generation approaches for XACML policies leverage combinatorial methodologies [9,10,11,12]. With the adoption of these methodologies, the generated number of test cases rapidly grows according to the policy complexity.

The execution of a large number of test cases can drastically increase the cost and effort of the testing phase especially for what concerns the oracle definition, i.e., checking the test results and deciding whether they are correct or not.

In practice, in the context of access control systems, except for some attempts to automatically derive the oracle from the XACML policy model [13], this oracle derivation is usually performed manually, because the complexity of the XACML language makes the use of automated support very difficult. Given the real constraints on testing budget, a key issue in the testing of XACML policies is to reduce as much as possible the number of tests to be executed while trying to maximize the fault detection effectiveness of the reduced test suite.

The main goal of this paper is to address this specific issue by proposing some testing coverage criteria specifically conceived on the peculiarities of the XACML 3.0 language that is the current standard language for access control policies definition. To define these XACML 3.0-based coverage criteria, we first provide a formal definition of the main elements of the XACML 3.0 language. The proposed XACML 3.0-based coverage criteria will guide the testing activities and enhance the trustworthiness of XACML-based access control systems.

As stated in the literature, coverage information can provide an indication of the effectiveness of the executed test cases and can guide the generation of test cases [14]. However, some empirical results [15] show that the performance of the reduced test suite could vary according to the considered program and the adopted coverage criterion.

In this paper, we provide some examples of how the proposed coverage criteria could be useful in addressing problems and inconsistencies in policy specification. In particular, we start from a formal definition of XACML syntax and semantics, and then, we show its use for enabling the understanding of XACML standard intentions and allow rigorous analysis techniques for verifiability and testability purposes.

Some attempts to provide formal reasoning techniques for the analysis and verification of policies have been provided [16,17,18,19,20]. They mainly focus on specific aspects of the language or design new expressive languages whose formal foundations enable tool-supported analysis and enforcement of access control policies.

In this paper, starting from the work of [16], we refine the definition of the abstract syntax and semantics of XACML 3.0 standard. According to this formal definition, we define a formal specification of the coverage process of the elements of the XACML policy and provide some coverage criteria that is useful to assess the XACML policy.

In particular, we select as the specification language the abstract syntax of the Context Free Grammar (CFG) [21], because it provides a precise mathematical definition that clearly rules out the XACML language. Additionally, the formalization provided by the CFG is a machine readable specification, which can be easily implemented in an automatic way.

Summarizing, the main contributions of this paper are: (i) to derive a rigorous formalization of XACML 3.0 standard leveraging context-free grammar [21], extending and revising the proposal of [16], and (ii) to formally define some policy coverage criteria for XACML 3.0 policy testing.

We also discuss possible ways to adopt the defined coverage criteria for improving the verifiability and testability of the policy such as: (i) the derivation of XACML requests according to the defined coverage criteria; (ii) the reduction (or selection) of a given test suite according to defined coverage criteria and its effect on fault detection; (iii) finally, the coverage measurement of the XACML policy in order to detect uncovered or redundant parts of the policy as well as guide the development of further test cases, which can improve the quality of policy testing.

The remainder of this paper is organized as follows. Section 2 introduces the XACML language. Section 3 presents the related work. Section 4 presents the formalization of XACML 3.0 policy elements, while Section 5 presents the formalization of the XACML request. Section 6 presents the coverage definition of XACML 3.0 policy elements, whereas Section 7 shows the definition of some coverage criteria and their application to a policy example. Finally, Section 8 concludes the paper, also hinting at future work.

## 2. Access Control Policies in XACML 3.0

XACML 3.0 language is a de facto standardized specification language for ruling the system access in an XML format. It relies on two main concepts: the XACML policy used for modeling the access requirements of a protected system; and the XACML request used for requiring the access to a protected resource. In the access control system, the request is evaluated against the policy to allow (or deny) the access to the resource. In the following, more details about these two concepts are provided.

An XACML policy is characterized by a tree structure having as its root the *PolicySet* and as children one (or more) *PolicySet*(s) or *Policy* elements. The latter includes: a *Target*, which specifies the execution constraints in terms of the subjects, resources, actions, and environments on which the policy can be applied; and a set of rules having also in turn a target, a condition and a rule-combining algorithm. Usually, the target is represented by a conjunctive sequence of *AnyOf* clauses. In particular, each *AnyOf* clause is a disjunctive sequence of *AllOf* clauses, and each *AllOf* clause is a conjunctive sequence of match predicates, and each match predicate compares attribute values in a request with the policy attributes. Both match predicates and rule conditions use different logical expressions and a variety of predefined functions and data types on subject, resource, action, and environment.

Only when a request satisfies the target of the *PolicySet* or *Policy*, the associated set of rules can be evaluated; otherwise, it is skipped.

Considering in detail the structure of a rule, its main elements are: the *Target* and the *Condition*, i.e., a set of Boolean functions used for establishing when the request is applicable to the rule. In this last case, the outcome of the rule is the rule effect (*Permit* or *Deny*). If the request is not applicable to the rule, the evaluation outcome is *NotApplicable* or *Indeterminate* in case of error.

The combining algorithms (either the *PolicyCombiningAlgorithm* or *RuleCombiningAlgorithm*) define how to combine the evaluation results in order to provide a unique evaluation outcome (access result). As an example, the *deny-overrides algorithm* establishes that *Deny* takes the precedence regardless of any other rules evaluation. Therefore, it will return *Deny* if there is a rule that is evaluated to *Deny*. It will return *Permit* if there is at least a rule that is evaluated to *Permit*, and all the other rules are evaluated to *NotApplicable*. Alternatively, an *Indeterminate* result is provided as the outcome if there is an error in the evaluation of a rule with the *Deny* effect, and the other policy rules with the *Deny* effect are not applicable.

Similarly, the *first-applicable* algorithm forces the rules evaluation in the order in which they are listed in the policy. The final outcome will be the effect of the first applicable rule (i.e., *Permit* or *Deny*). In all the cases, if the evaluation of the rule target (or the rule condition) is *False*, the next rule in the order will be evaluated until no further rule in the order exists, and the final *NotApplicable* result is provided.

For the evaluation, all attribute and element values describing the subject, resource, action, and environment of an access request are considered and compared with the attribute and element values of the policy.

**Figure 1:**
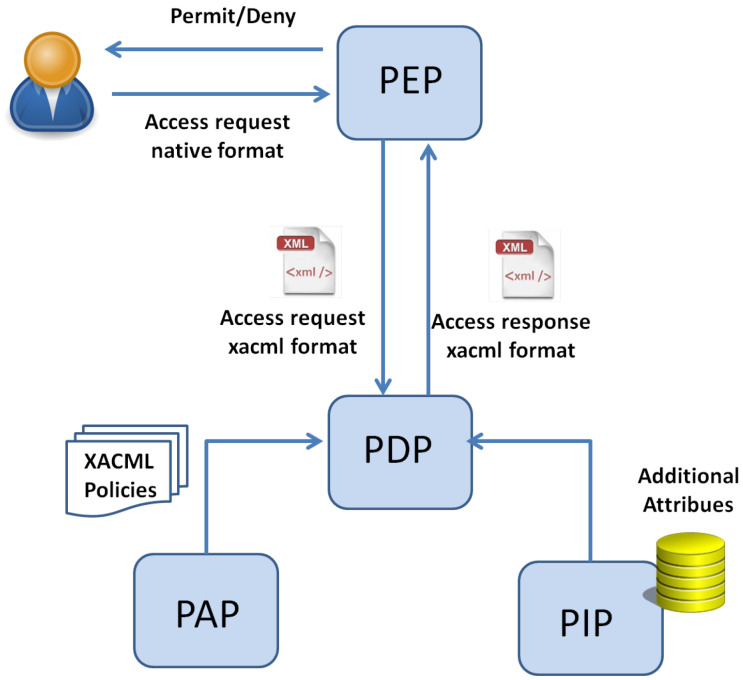
XACML Basic Model.

The above-described access control mechanism relies on the standardized access control system architecture represented in Figure 1. As in the Figure, the main entities are:

- the Policy Enforcement Point (PEP) that is in charge of receiving the user’s request, transforming it into an XACML request and sending it to the PDP. It also allows or denies the access to the resource.
- the Policy Decision Point (PDP) which is in charge of the request evaluation against the policy and the computation of the access response (Permit/Deny/NotApplicable). The PDP retrieves the access policies from the Policy Administration Point (PAP) and the attributes values from the Policy Information Point (PIP).

## 3. Related Work

This work spans over two main research directions: XACML formalization (Section 3.1) and coverage criteria for testing purposes (Section 3.2). Moreover, in Section 3.3, the specific advancements of our work with respect to the SotA are presented.

### 3.1. XACML Formalization

Access control languages formalization has recently been the subject of extensive research, and several attempts have been provided to analyze and formalize XACML policies.

Margrave is a popular propositional logic analysis tool for XACML policy verification [22]. Logic programming systems and description logics (DL) have been used to model XACML policy constraints and roles hierarchies [23,24]. Specifically, the work in [24] extends the analysis services offered by Margrave leveraging full First-Order logic XACML analysis tools (such as Alloy [25]). It is able to cover the analysis of role hierarchies and role cardinality as well as analysis of policy redundancy.

Bryans explores the use of process algebra for formalizing and analyzing XACML language, presenting the core concepts of XACML using Communicating Sequential Processes (CSP) [26]. Other woks propose different formalization approaches for capturing the semantics of XACML constructs [27,28,29] and specifying an access control meta-model to derive access control policies or rule-based policies [30,31,32].

Other approaches to enable the automated verification of access control policies rely on SAT solver. Specifically, the authors of [33] present a formal model defining the semantics of the XACML access control language; then, they define ordering relations on access control policies that are used to automatically verify properties of the policies by translating them to boolean satisfiability problems and then applying a sat solver.

Masi et al. in [17] define a formal expressive access control policy language denoted as FACPL, supporting automated specification, analysis, and the enforcement of access control policies. It refines and extends XACML language and relies on a rigorously defined denotational semantics that allows for the management of missing attributes and formalization of combining algorithms. For enabling automated policies analysis, they introduce a constraint formalism based on Satisfiability Modulo Theories (SMT) formulae, which has been proven to be more effective than other ones, such as decision diagrams or description logic.

A more recent work [13] aims to formalize XACML policies by using a typed graph, called the XAC-Graph. Then, this XAC-Graph is used for both XACML requests generation and automated model-based oracle derivation.

Finally, the work in [16] differently from the previous approaches focuses on the last standard version of XACML that is XACML 3.0 and provides a formally defined semantics for XACML 3.0 components evaluation and standard combining operators, proposing new directions for the XACML standard extension.

Our proposal revises and extends the work of [16], proving a complete definition of the abstract syntax and semantics of XACML 3.0 standard, leveraging context-free grammars. Moreover, differently from all the mentioned formalization approaches, our work focuses more on policy testing than the verification of policies properties. For this, we use the proposed formalization to define some policy coverage criteria that can enhance the effectiveness of policy testing activity.

### 3.2. Coverage-Based Policy Testing

Coverage metrics represent an effective way to assess the test quality and compare different testing solutions. The aim of adequacy criteria is to evaluate a specific testing strategy by measuring the percentage of the exercised elements in the program or in its specification.

In the literature, test coverage is adopted for different purposes: (i) enhancing the test suite with additional test cases in order to exercise elements that have not been tested; (ii) test set augmentation and test set minimization in the context of regression testing [34]; (iii) selection of test cases and evaluation of test cases effectiveness [35]; (iv) test cases prioritization [36], aiming to reorder test cases so that the tests with a higher priority can be executed before those with a lower priority; and finally, (v) software maintenance [34]. The authors of [34] propose a systematic survey of coverage-based testing, whereas the work in [37] focuses on coverage criteria for state-based testing.

Many solutions for test coverage measurement and analysis have been proposed. They are based on the adopted policy specification language. The work in [38] provides a first attempt of coverage criterion for XACML policies. It defines three different structural coverage metrics able to reduce the generated test sets and validates the effects of this test’s reduction in terms of fault detection.

An extension of the coverage solution presented in [38] is proposed in [39]. This work defines an XACML-based smart coverage selection approach that focuses on the policy rules coverage and provides a formalization of the proposed coverage criterion relying on the *Rule Target Set* concept, i.e., the union of the target of the rule, and all enclosing policy and policy sets targets. The main concept of the proposed criterion is that in order to match the rule target, the requests must first match the enclosing policy and policy sets targets.

Cirg (Change-Impact Request Generation) [40] is for instance a framework able to generate access requests through the change-impact analysis of two synthesized versions of an XACML policy and allows a reduction of the number of tests based on policy structural coverage. The work in [41] leverages mutation analysis and coverage analysis to perform regression testing of security policies.

More recently, in [12], a family of coverage criteria for XACML policies, including Modified Condition/Decision Coverage (MC/DC), has been presented and evaluated through mutation analysis in order to establish the most effective one in terms of fault detection. In [42], a proposal for the continuous tracing of policy execution and corresponding coverage criteria has been presented in order to detect inconsistencies in the policy specification and provide support for policies updates if new events occur.

Differently from previous solutions, our proposal provides a rigorous formalization of coverage of XACML 3.0 elements and formally specifies four additional coverage criteria specifically conceived for XACML 3.0 policy testing purposes.

### 3.3. SotA Advancements

In order to clarify the position of our paper with respect to the State of the Art, we report the analysis of the related works in the last 10 years in Table 1. In particular, in the first column, we provide the reference of the paper and the publication year; in the column labeled *Paper contribution*, we summarize the main contribution of the considered related work; in the column labeled *Language/Formalism*, we report the target specification language; in the columns labeled *Access control formalization* and *Coverage metrics/ measures*, we specify if the paper focuses on the formalization or on the coverage metrics and measures, respectively. Finally, in the last column labeled *Our advancement with respect to SotA*, we provide the advancements of our paper with respect to the considered related work. In the last row of the table, we provide the classification of our contribution.

As shown in the table, most of the related works focus on the possible formalization of the access control policy and its constructs either considering XACML or ABAC/RBAC specification language. Coverage criteria and metrics have been rarely analyzed or improved during these last 10 years.

As evidenced by the table, the related works are strictly divided into two categories: either they focus on the formalization or they provide coverage criteria. To the best of our knowledge, as evidenced in the last row of the table, our paper is the first attempt to combine the two categories with the purpose of providing a comprehensive process useful for both developers and testers.

## 4. Formalization of Primary XACML 3.0 Elements

In this section, we provide the formalization of the XACML standard for describing security access control policies. Starting from the seminal work of [16], we provide here the definition of the abstract syntax of XACML 3.0 standard (Section 4.1), the specification of the XACML grammar (Section 4.2), and an example of policy formalization (Section 4.3).

### 4.1. XACML 3.0 Primary Elements

In this section, we introduce the alphabet of the grammar we use to describe the structure of an XACML access control policy. In particular, we extend and revise the XACML syntax introduced in [16], so to better represent the coverage concepts of this paper. For aim of completeness, we report in Table 2 the symbols we use in this paper for the definition of the grammar.

An initial formalization of the syntactical categories is the one illustrated in Table 3. It focuses on the syntactical sets that can be identified in an XACML access control specification and uses the Kleene star operator for deciding whether a string belongs to a grammar or not.

According to this formalization, given a policy P having a set of *n* rules $R=\{R_{1},\ldots ,R_{n}\}$, and a Policy target $T_{P}$ possibly empty, the Boolean satisfiability (SAT) of the policy can be defined as:

$$\left\{\begin{array}{cc} \left(P=\{R\})\Rightarrow SAT(R\right) & \mathrm{if}T_{P}=\epsilon \\ \left(P=\left\{T_{P},R\right\}\right)\Rightarrow \left(SAT\left(T_{P}\right)\Rightarrow SAT\left(R\right)\right) & \mathrm{otherwise} \end{array}\right.$$

In the similar way, the Boolean satisfiability (SAT) of each of the policy rules, generically indicated with R, can be defined as:

$$\left\{\begin{array}{cc} \left(R=\emptyset \right)\Rightarrow t_{\mathrm{rue}} & \\ \mathrm{if}T_{R}=\epsilon \wedge C=\epsilon \\ \left(R=\left\{C\right\}\right)\Rightarrow \left(SAT\left(T_{P}\right)\Rightarrow eval\left(C\right)\right) & \\ \mathrm{if}T_{R}=\epsilon \\ \left(R=\left\{T_{R}\right\}\right)\Rightarrow SAT\left(T_{R}\right) & \\ \mathrm{if}C=\epsilon \\ \left(R=\left\{T_{R}\right\}\cup \left\{C\right\}\right)\Rightarrow \left(SAT\left(T_{R}\right)\Rightarrow eval\left(C\right)\right) & \mathrm{otherwise} \end{array}\right.$$

### 4.2. XACML 3.0 Grammar

Formally, a context-free grammar [21] is defined by a quadruple:

G=〈Λ,V,S,P〉

where

- Λ is the set of symbols, called alphabet;
- *V* is the finite set of the syntactical categories;
- *S* is the main syntactical category;
- *P* is the set of all productions, where each production, in the case of context-free grammars (which is the one we need for XACML 3.0) is a finite relation:
  A→α
  where $A\in V,\;\alpha \in {\left(\Lambda \cup V\right)}^{*}$ (the asterisk represents the Kleene star).
  Applying the above definition to the XACML 3.0 access control language, we have:
  the set Λ is a subset of all the possible elements of the XACML 3.0, and it is defined as
  $$\begin{array}{c} \Lambda =V_{0}\cup \{a_{v},a_{s},a_{d},s,r,a,e,a_{h},p_{ermit},d_{eny},t_{rue},f_{alse},\\ p_{ermit}o_{verrides},d_{eny}o_{verrides},f_{irst}a_{pplicable},\\ o_{nly}o_{ne}a_{pplicable},d_{eny}u_{nless}p_{ermit},p_{ermit}u_{nless}d_{eny},\\ o_{rdered}d_{eny}o_{verrides},o_{rdered}p_{ermit}o_{verrides}\} \end{array}$$

where

$$\begin{array}{c} V_{0}=\{P_{S},T_{PS},P,P_{CA},T_{P},A_{nyOf},A_{llOf},M,A_{ttrChoice},\\ A_{ttr},R,R_{CA},T_{R},C,{E_{X}}_{bool},A,E_{X},{E_{X}}_{fun},F_{un},\\ E_{ffect},S_{ubject},R_{esource},A_{ction},E_{nv},A_{dHoc}\} \end{array}$$

The main syntactical category *S* is defined as:

$$S=\{P_{S}\}$$

The set of all productions *P* is defined in Table 4.

### 4.3. Example of Policy and Its Formalization

In this section, we provide an example of policy representation through the grammar introduced in the previous section. The sample policy considered is shown in Listing 1. In this case, starting from S={PS}, the policy can be expressed by the following production:

1–160 In Listing 1, the PolicySet element contains: two Policy elements (Policy1 and Policy2 at line 30 and line 96 of Listing 1 respectively), the declaration of the policy-combining algorithm considered that in this case is first applicable (line 8), and a policy set Target element (lines 12–29 of Listing 1) that specifies that the subject element must be an integer less than 15. This can be expressed as:
  PS→PSP2→TPSPCAP1P2→AnyOfFirstApplicableP1P2→AllOfFirstApplicableP1P2→MFirstApplicableP1P2→FunavAttrChoiceFirstApplicableP1P2→integerlessthanavasFirstApplicableP1P2
30–95 Each of the policies in the policy set has in turn a Target element and a Rule element. In particular, Policy1 specifies that the rule-combining algorithm considered is first applicable (line 32), the policy Target element (lines 39–56 of Listing 1) specifies that the subject element must be equal to the integer 10. This can be expressed as:
  P1→RCATP1R1→FirstApplicableAnyOfR1→FirstApplicableAllOfR1→FirstApplicableMR1→FirstApplicableFunavAttrChoiceR1→FirstApplicableintegerequalavasR1
57–94 The Rule1 element returns the Deny effect (line 58) in case: (i) the subject element is greater than 4 as declared in the rule Target (lines 59–77 of Listing 1) and (ii) the subject element is greater than 2 as declared in the rule Condition (lines 78–93 of Listing 1). This can be expressed as:
  R1→EffectTC→DenyAnyOfC→DenyAllOfC→DenyMC→DenyFunavAttrChoiceC→Deny integergreaterthan av adC→Deny integergreaterthan av adEXbool→Deny integergreaterthan av adA→Deny integergreaterthan av adAEX→Deny integergreaterthan av adAEXad→Deny integergreaterthan av adEXfunav ad→Deny integergreaterthan av ad integergreaterthan av ad
96–159 Similarly, Policy2 specifies that the rule-combining algorithm considered is first applicable (line 97), whereas the Target element (lines 104–121 of Listing 1) specifies that the subject element must be less than the integer 9 and there is a Rule element. This can be expressed as:
  P2→RCATP2R2→FirstApplicableAnyOfR2→FirstApplicableAllOfR2→FirstApplicableMR2→FirstApplicableFunavAttrChoiceR2→FirstApplicableintegerlessthanavasR2
122–158 The Rule2 element returns the Permit effect (line 123) in case: (i) the subject element is less than 7 as declared in the rule Target (lines 124–142 of Listing 1) and (ii) the subject element is less than 3 as declared in the rule Condition (lines 143–157 of Listing 1). This can be expressed as:
  R2→EffectTC→PermitAnyOfC→PermitAllOfC→PermitMC→PermitFunavAttrChoiceC→Permit integerlessthan av adC→Permit integerlessthan av adEXbool→Permit integerlessthan av adA→Permit integerlessthan av adAEX→Permit integerlessthan av adAEXav→Permit integerlessthan av adAEXfunadav→Permit integerlessthan av ad integerlessthan ad av

Basically, if we see the parsing procedure of this policy as an automaton, we obtain:




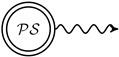



    integerlessthanavasFirstApplicable    FirstApplicableintegerequalavasDenyintegergreaterthanavadintegergreaterthanavadFirstApplicableintegerlessthanavasPermitintegerlessthanavadintegerlessthanadav




In addition, for this example, the parsing procedure returns a positive result, that is, the policy is fully covered by the grammar; this means that the policy is syntactically correct.
**Listing 1.** XACML Policy Example.

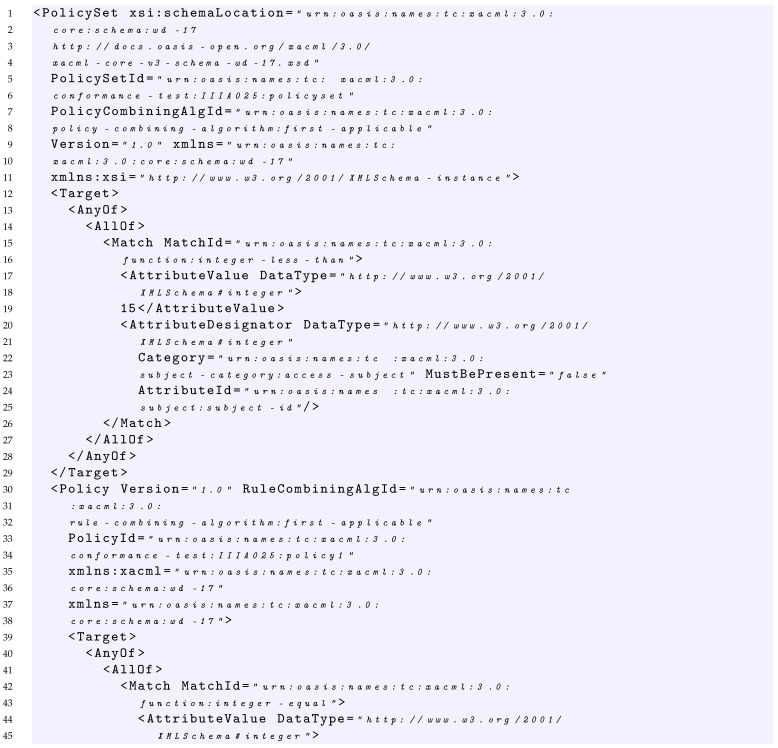


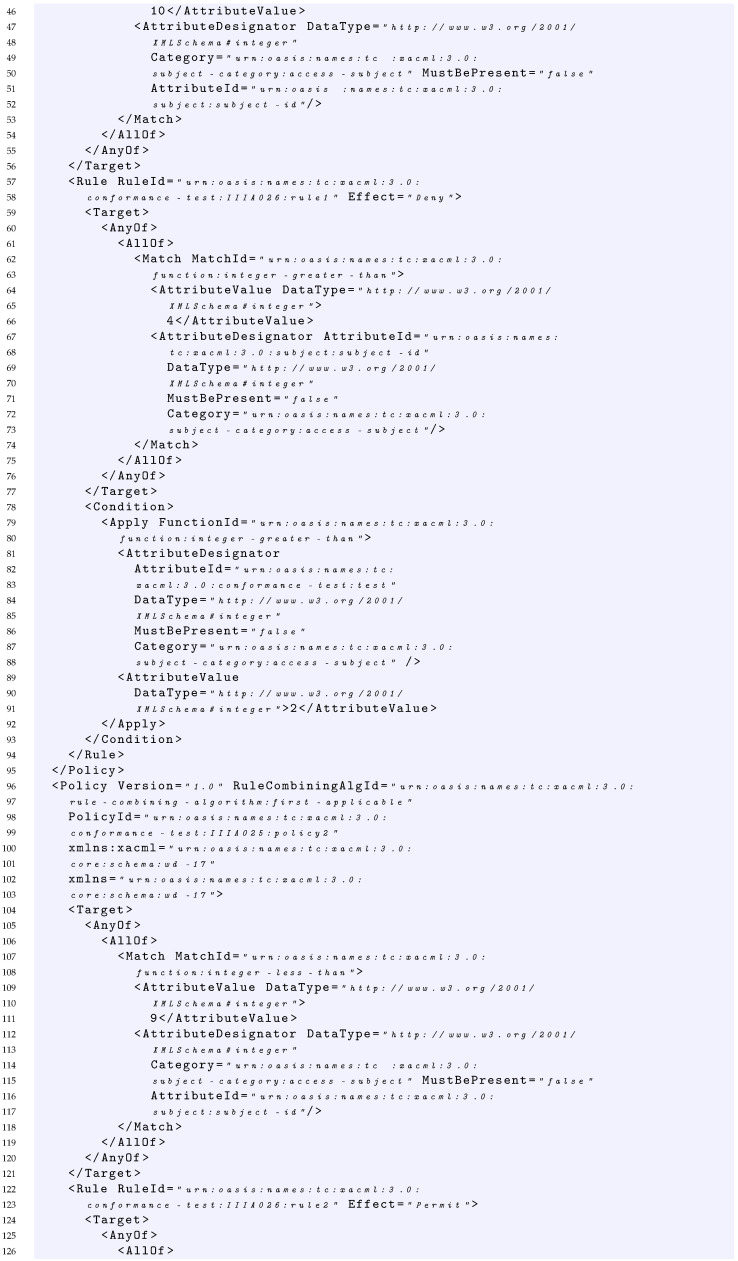


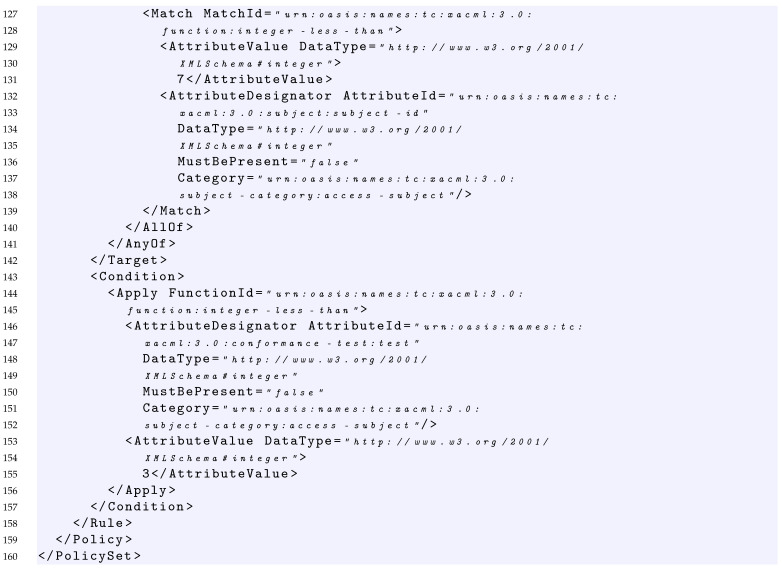


## 5. Definition of the Request Structure

**Figure 2:**
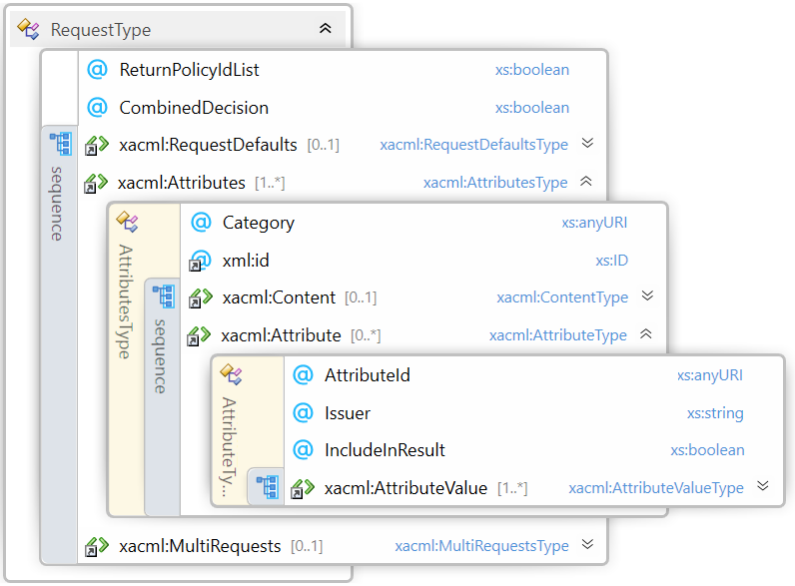
Request structure.

In this section, we provide the formalization of the XACML request information (such as attribute values, ID, and so on) necessary for being evaluated by a policy. In particular, in Figure 2, the generic request structure is represented, which contains:

- AttributeValue element defined by a DataType and any attribute.
- Attribute element A is composed by $\{\mathrm{AttributeID},{\mathrm{AV}}_{1},\ldots ,{\mathrm{AV}}_{n}\},n\geq 1$, where $\forall i,{\mathrm{AV}}_{i}$ is an AttributeValue and the AttributeID is the identifier of the attribute.
- Attributes element A is defined by an AttributeID, a Category and a set of Attribute (that can also be empty), so basically:
  $$A=\{\mathrm{AttributeID},\mathrm{Category},A_{1},\ldots ,A_{n}\},n\geq 0$$
- Request element: R has this structure: $\{A_{1},\ldots ,A_{n}\},n\geq 1$ where $\forall i,A_{i}$ is an Attributes element.

In Listings 2 and 3, two examples of requests relative to the policy of Listing 1 are provided.
**Listing 2.** XACML Request1 Example. 
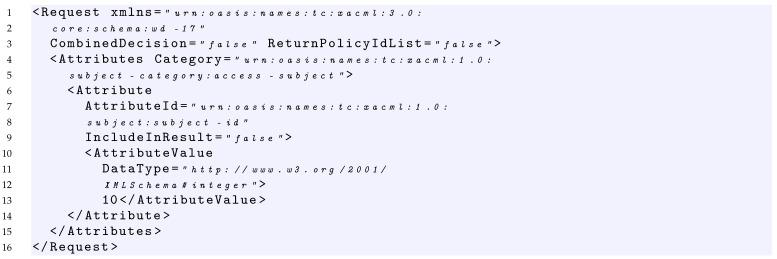

**Listing 3.** XACML Request2 Example.

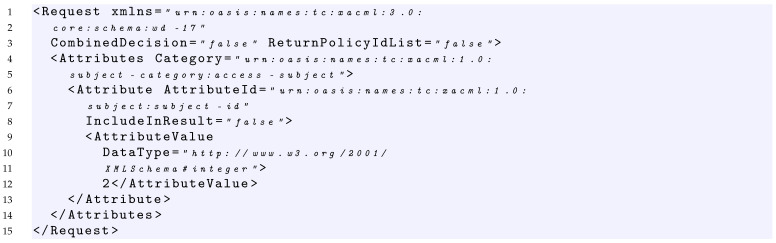



## 6. XACML-Based Coverage Definition

In this section, we detail the concepts of coverage of the XACML 3.0 grammar elements. Considering therefore a request R and an element of XACML 3.0 grammar, the following coverage function can be introduced.

**Definition** **1** (Coverage function). *Let $el_{XACML}$ be an* XACML 3.0 *component and R a* *request* *element, such that $R=\{A_{1},\ldots ,A_{n}\}$*

*We define the function*

$$\mathrm{cov}:el_{\mathrm{XACML}}\times R\to b$$

*where $b\in \{T_{\mathrm{rue}},F_{\mathrm{alse}}\}$.*

In practice, this function establishes if the request can be evaluated or not by the considered element.

Following a more intuitive way of coverage analysis, in the remainder of this section, we provide the coverage of each of the grammar elements starting from the basic ones.

### 6.1. Covering Match and Condition

Let $M=f_{\mathrm{MatchID}}\left(v,c,a\right)$ be a Match element where $f_{\mathrm{MatchID}}$ is an identifier of the Match function, *v* is the embedded AttributeValue, *c* is the attribute Category related to *v*, and *a* is an AttributeChoice (defined before, that can be mutually or exclusively, an AttributeDesignator or an AttributeSelector). Covering the Match element with a request $R=\{A_{1},\ldots ,A_{n}\}$ is defined as follows:

(1) $$\mathrm{cov}\left(f_{\mathrm{MatchID}}\left(v,c,a\right),R\right)=\left\{\begin{array}{cc} T_{\mathrm{rue}} & \mathrm{if}\;\exists i:v^{\prime },c^{\prime }\in A_{i}.\\ & f_{\mathrm{MatchID}}\left(v^{\prime },c^{\prime },a\right)=T_{\mathrm{rue}}\\ F_{\mathrm{alse}} & \mathrm{if}\forall i:\forall v^{\prime },c^{\prime }\in A_{i}.\\ & f_{\mathrm{MatchID}}\left(v^{\prime },c^{\prime },a\right)=F_{\mathrm{alse}} \end{array}\right.$$

Note that in the definition, $f_{\mathrm{MatchID}}$ appears twice: the former one for the syntax element of Match component, while the latter indicates the coverage of the $f_{\mathrm{MatchID}}$ function.

In this case, considering the request of Listing 2 which has a subject with value 10, it is able to cover the $f_{\mathrm{MatchID}}$ of the Policy Set of Listing 1.

Considering instead the coverage of the condition element, let C be a Condition and R be a request; then, it is possible to define:

$$\mathrm{cov}\left(C,R\right)=\left\{\begin{array}{c} T_{\mathrm{rue}}\\ F_{\mathrm{alse}} \end{array}\right.$$

Considering the policy of Listing 1 and the request of Listing 2, the request covers the condition expressed in the first rule with the value true because it contains a value greater than 2.

### 6.2. Covering AllOf, AnyOf and Target

Let M be a Match, A a AllOf, E a AnyOf, and T a Target.

Suppose that $\mathrm{allof}\left(A_{id}\right):\left\{M_{1},\ldots ,M_{n}\right\},n\geq 1$ is an AllOf element and $\forall i,M_{i}$ is a Match element. The coverage of AllOf element $A_{id}$ over a request R is as follows:

$$\mathrm{cov}\left(A_{id},R\right)=\left\{\begin{array}{cc} T_{\mathrm{rue}} & \mathrm{if}\forall i,1\leq i\leq n:\mathrm{cov}\left(M_{i},R\right)=T_{\mathrm{rue}}\\ F_{\mathrm{alse}} & \mathrm{if}\exists i,1\leq i\leq n:\mathrm{cov}\left(M_{i},R\right)=F_{\mathrm{alse}} \end{array}\right.$$

Suppose that $\mathrm{anyof}\left(E_{id}\right):\left\{A_{1},\ldots A_{n}\right\},n\geq 1$ is an AnyOf element and $\forall i,A_{i}$ is a AllOf element. The coverage of AnyOf element $E_{id}$ over a RequestR is defined as follows:

$$\mathrm{cov}\left(E_{id},R\right)=\left\{\begin{array}{cc} T_{\mathrm{rue}} & \mathrm{if}\exists i,1\leq i\leq n:\mathrm{cov}\left(A_{i},R\right)=T_{\mathrm{rue}}\\ F_{\mathrm{alse}} & \mathrm{if}\forall i,1\leq i\leq n:\mathrm{cov}\left(A_{i},R\right)=F_{\mathrm{alse}} \end{array}\right.$$

Suppose that $\mathrm{target}\left(T_{id}\right):\left\{E_{1},\ldots ,E_{n}\right\},n\geq 0$ is a Target element and $\forall i,E_{i}$ is an AnyOf element. The coverage of Target $T_{id}$ over a Request R is defined with the following equation:

$$\mathrm{cov}\left(T_{id},R\right)=\left\{\begin{array}{cc} T_{\mathrm{rue}} & \mathrm{if}\left(n=0\right)\vee (\forall i,1\leq i\leq n:\\ & \mathrm{cov}\left(E_{i},R\right)=T_{\mathrm{rue}})\\ F_{\mathrm{alse}} & \mathrm{if}\exists i,1\leq i\leq n:\mathrm{cov}\left(E_{i},R\right)=F_{\mathrm{alse}} \end{array}\right.$$

Considering the policy of Listing 1, the target of *Rule1* and the request of Listing 2, the request covers the AllOf, the AnyOf and the *Target* elements of this rule with the values true. Indeed, the value 10 of the request matches the function string equal of the AllOf element, and because the AnyOf and the Target contain only an element, consequently, the request covers also these last.

### 6.3. Covering Rule

Let R be a Rule, T a Target, C a Condition, E an Effect (with possible values: permit and deny).

Suppose that rule(R)={T,C,E}. The coverage of Rule element $R_{id}$ over a request R is determined as follows:

$$\mathrm{cov}\left(R_{id},R\right)=\left\{\begin{array}{cc} T_{\mathrm{rue}} & \mathrm{if}\left(\mathrm{cov}\left(T,R\right)=T_{\mathrm{rue}}\;\vee \;T=\epsilon \right)\wedge \\ & \left(\mathrm{cov}\left(C,R\right)=T_{\mathrm{rue}}\;\vee \;C=\epsilon \right)\\ F_{\mathrm{alse}} & \mathrm{if}\mathrm{cov}\left(T,R\right)=F_{\mathrm{alse}}\vee \\ & \left(\mathrm{cov}\left(T,R\right)=T_{\mathrm{rue}}\wedge \right.\\ & \left.\mathrm{cov}\left(C,R\right)=F_{\mathrm{alse}}\right) \end{array}\right.$$

Considering the policy of Listing 1 and the request of Listing 2, the request covers the Rule1 since it covers with a true value both the Target and Condition of Rule1.

### 6.4. Covering Policy

Let P be a Policy, T a Target, $\langle R_{1},\ldots R_{n}\rangle$ a sequence of Rules and ${\mathrm{comb}}_{id}$ a Combining Algorithm.

Suppose that $\mathrm{policy}\left(P_{id}\right)=\left\{T,\langle R_{1},\ldots R_{n}\rangle ,{\mathrm{comb}}_{id}\right\}$.

We define with R the coverage over a request R of the sequence of Rules:

$$R=\langle \mathrm{cov}\left(R_{1},R\right),\ldots \mathrm{cov}\left(R_{n},R\right)\rangle$$

Then, the coverage of Policy $P_{id}$ over a request R is defined as follows:

$$\mathrm{cov}\left(P_{id},R\right)=\left\{\begin{array}{cc} T_{\mathrm{rue}} & \mathrm{if}\mathrm{cov}\left(T,R\right)=T_{\mathrm{rue}}\;\wedge \\ & comb_{id}\left(R\right)=T_{\mathrm{rue}}\\ F_{\mathrm{alse}} & \mathrm{if}\mathrm{cov}\left(T,R\right)=F_{\mathrm{alse}}\;\vee \\ & \left(\mathrm{cov}\left(T,R\right)=T_{\mathrm{rue}}\;\wedge \right.\\ & \left.comb_{id}\left(R\right)=F_{\mathrm{alse}}\right) \end{array}\right.$$

The coverage of the sequence of Rules, defined by R, depends on the chosen Combining Algorithm $comb_{id}$. The semantic within the combining algorithm bound to the coverage of R over a request R can be formally defined is this way:

- If $comb_{id}=f_{a}$, then the algorithm that must be applied is the *first-applicable*:
  $$f_{a}\left(R\right)=\left\{\begin{array}{cc} T_{\mathrm{rue}} & \mathrm{if}\exists i,1\leq i\leq n:\\ & \mathrm{cov}\left(R_{i},R\right)=T_{\mathrm{rue}}\wedge \\ & \forall j,1\leq j<i:\mathrm{cov}\left(R_{j},R\right)=F_{\mathrm{alse}}\\ F_{\mathrm{alse}} & \mathrm{if}\forall i,1\leq i\leq n:\mathrm{cov}\left(R_{i},R\right)=F_{\mathrm{alse}} \end{array}\right.$$
- If $comb_{id}=oo_{a}$, then the algorithm that must be applied is the *only-one-applicable*:
  $$oo_{a}\left(R\right)=\left\{\begin{array}{cc} T_{\mathrm{rue}} & \mathrm{if}\exists i,1\leq i\leq n:\\ & \mathrm{cov}\left(R_{i},R\right)=T_{\mathrm{rue}}\wedge \\ & \left(\forall j,1\leq j\leq n:i\neq j\wedge \right.\\ & \left.\mathrm{cov}\left(R_{j},R\right)=F_{\mathrm{alse}}\right)\\ F_{\mathrm{alse}} & \mathrm{if}\forall i,1\leq i\leq n:\mathrm{cov}\left(R_{i},R\right)=F_{\mathrm{alse}} \end{array}\right.$$
- If $comb_{id}=p_{o}$, then the algorithm that must be applied is the *permit-overrides*:
  $$p_{o}\left(R\right)=\left\{\begin{array}{cc} T_{\mathrm{rue}} & \mathrm{if}\exists i,1\leq i\leq n:\mathrm{cov}\left(R_{i},R\right)=T_{\mathrm{rue}}\wedge \\ & E_{R_{i}}=\mathrm{permit}\\ F_{\mathrm{alse}} & \mathrm{if}\forall i,1\leq i\leq n:\mathrm{cov}\left(R_{i},R\right)=F_{\mathrm{alse}}\vee \\ & \left(\exists i,1\leq i\leq n:\mathrm{cov}\left(R_{i},R\right)=T_{\mathrm{rue}}\wedge \right.\\ & \left.E_{R_{i}}=\mathrm{deny}\right) \end{array}\right.$$
- If $comb_{id}=d_{o}$ then the algorithm that must be applied is the *deny-overrides*:
  $$d_{o}\left(R\right)=\left\{\begin{array}{cc} T_{\mathrm{rue}} & \mathrm{if}\exists i,1\leq i\leq n:\mathrm{cov}\left(R_{i},R\right)=T_{\mathrm{rue}}\wedge \\ & E_{R_{i}}=\mathrm{deny}\\ F_{\mathrm{alse}} & \mathrm{if}\forall i,1\leq i\leq n:\mathrm{cov}\left(R_{i},R\right)=F_{\mathrm{alse}}\vee \\ & \left(\exists i,1\leq i\leq n:\mathrm{cov}\left(R_{i},R\right)=T_{\mathrm{rue}}\wedge \right.\\ & \left.E_{R_{i}}=\mathrm{permit}\right) \end{array}\right.$$

Considering the policy of Listing 1 and the request of Listing 2, the request covers Policy1, since it covers with true value both the Target of the policy and the first applicable rule that is Rule1.

### 6.5. Covering PolicySet

Let PS be a PolicySet, ${\mathrm{comb}}_{id}$ a Combining Algorithm, T a target, $\langle P_{1},\ldots ,P_{n}\rangle$ a sequence of Policy and PolicySet. Suppose that

$$\mathrm{policyset}\left({\mathrm{PS}}_{id}\right)=\left\{{\mathrm{comb}}_{id},T,\langle P_{1},\ldots ,P_{n}\rangle \right\}$$

We define P as the coverage over a request R of the sequence of Policy and PolicySet:

$$P=\langle \mathrm{cov}\left(P_{1},R\right),\ldots ,\mathrm{cov}\left(P_{n},R\right)\rangle$$

Then, the covering of PolicySet ${\mathrm{PS}}_{id}$ is defined as follows:

$$\mathrm{cov}\left({\mathrm{PS}}_{id},R\right)=\left\{\begin{array}{cc} T_{\mathrm{rue}} & \mathrm{if}\mathrm{cov}\left(T,R\right)=T_{\mathrm{rue}}\wedge \\ & comb_{id}\left(P\right)=T_{\mathrm{rue}}\\ F_{\mathrm{alse}} & \mathrm{if}\mathrm{cov}\left(T,R\right)=F_{\mathrm{alse}}\vee \\ & \left(\mathrm{cov}\left(T,R\right)=T_{\mathrm{rue}}\wedge \right.\\ & \left.comb_{id}\left(P\right)=F_{\mathrm{alse}}\right) \end{array}\right.$$

We do not formally provide here the definition of policy-combining algorithm because the semantic can be easily deduced from that already provided for the rule-combining algorithm over a request presented in the previous section.

Considering the policy of Listing 1 and the request of Listing 2, the request covers the PolicySet, since it covers with a true value both the Target of the PolicySet and the first applicable policy that is Policy1.

## 7. Coverage Criteria

Considering the coverage concepts introduced in the previous section, several criteria can be defined for testing purposes. In this section, we introduce some of them considering different ways in which a target and a condition element could be exercised during the requests evaluation. Specifically, some basic coverage criteria are: either having all the policy Target elements covered with the *True* value or having all the policy Target elements covered with the *True* and *False* value. Similarly, other coverage criteria are: either having all the policy Condition elements covered with the *True* value or having all the policy Condition elements covered with the *True* and *False* value. From a more formal point of view, this can be translated into:

- Policy targets true:
  Let RQ be a set of requests $\langle {\mathrm{RQ}}_{1},\ldots {\mathrm{RQ}}_{m}\rangle$, a
  $$\mathrm{policyset}\left(\mathrm{PS}\right)=\left\{{\mathrm{comb}}_{\mathrm{ps}},\mathrm{TPS},\langle P_{1},\ldots ,P_{n}\rangle \right\}$$
  where ${\mathrm{comb}}_{ps}$ is the Combining Algorithm, TPS is the target of the policy set, and $\langle P_{1},\ldots ,P_{n}\rangle$ a sequence of Policy elements. Let each of the $P_{\mathrm{id}}$ be a $\mathrm{policy}\left(P_{\mathrm{id}}\right)=\left\{{\mathrm{TP}}_{\mathrm{id}},\langle R_{1},\ldots R_{h}\rangle ,{\mathrm{comb}}_{p}\right\}$ where ${\mathrm{TP}}_{\mathrm{id}}$ is the Target of the policy, $\langle R_{1},\ldots R_{h}\rangle$ is the set of Rules and ${\mathrm{comb}}_{p}$ is the Combining Algorithm. Finally, let each $R_{\mathrm{id}}$ be a $\mathrm{rule}\left(R_{\mathrm{id}}\right)=\left\{{\mathrm{TR}}_{\mathrm{id}},C,E\right\}$ where ${\mathrm{TR}}_{\mathrm{id}}$ is the Target of the rule, C is the Condition, and E is the Effect.
  The set of requests RQ covers the criterion of the Policy targets true if for each of the target elements included in the policy PS ($\mathrm{TPS},{\mathrm{TP}}_{\mathrm{id}},{\mathrm{TR}}_{\mathrm{id}}$) there exists at least an ${\mathrm{RQ}}_{i}$ that covers the target with the true value.
- Policy targets true and false.
  Let RQ be a set of requests $\langle {\mathrm{RQ}}_{1},\ldots {\mathrm{RQ}}_{m}\rangle$ a
  $$\mathrm{policyset}\left(\mathrm{PS}\right)=\left\{{\mathrm{comb}}_{\mathrm{ps}},\mathrm{TPS},\langle P_{1},\ldots ,P_{n}\rangle \right\}$$
  where ${\mathrm{comb}}_{ps}$ is the Combining Algorithm, TPS is the target of the policy set, and $\langle P_{1},\ldots ,P_{n}\rangle$ is a sequence of Policy elements. Let each of the $P_{\mathrm{id}}$ be a $\mathrm{policy}\left(P_{\mathrm{id}}\right)=\left\{{\mathrm{TP}}_{\mathrm{id}},\langle R_{1},\ldots R_{h}\rangle ,{\mathrm{comb}}_{p}\right\}$ where ${\mathrm{TP}}_{\mathrm{id}}$ is the Target of the policy, $\langle R_{1},\ldots R_{h}\rangle$ is the set of Rules and ${\mathrm{comb}}_{p}$ is the Combining Algorithm. Finally, let each $R_{\mathrm{id}}$ be a $\mathrm{rule}\left(R_{\mathrm{id}}\right)=\left\{{\mathrm{TR}}_{\mathrm{id}},C,E\right\}$ where ${\mathrm{TC}}_{\mathrm{id}}$ is the Target of the rule, C is the Condition, and E is the Effect.
  The set of requests RQ covers the criterion of the Policy targets true and false if for each of the target element included in the policy PS ($\mathrm{TPS},{\mathrm{TP}}_{\mathrm{id}},{\mathrm{TR}}_{\mathrm{id}}$), there exists at least an ${\mathrm{RQ}}_{i}$ that covers the target with the true value and at least an ${\mathrm{RQ}}_{j}$ that covers the target with the false value.
  According to the target coverage defined in the previous section, if a Target element is empty, it is always covered with $T_{\mathrm{rue}}$. From a practical point of view, the *True* evaluation of a Target element requires a request that makes at least one element AnyOf to *True*; in case of the AnyOf element of the AllOf elements, a request is necessary that makes all the Match elements *True* (in our analysis, the Match elements are the leaves of the tree with the Policy or PolicySet component as the root).
  In a similar way, two additional criteria that refine the previous ones can be defined. They focus on the evaluation of the condition and can be formulated in the following.
- Policy conditions true:
  Let RQ be a set of requests $\langle {\mathrm{RQ}}_{1},\ldots {\mathrm{RQ}}_{m}\rangle$, a
  $$\mathrm{policyset}\left(\mathrm{PS}\right)=\left\{{\mathrm{comb}}_{\mathrm{ps}},\mathrm{TPS},\langle P_{1},\ldots ,P_{n}\rangle \right\}$$
  where ${\mathrm{comb}}_{ps}$ is the Combining Algorithm, TPS is the target of the policy set and $\langle P_{1},\ldots ,P_{n}\rangle$ is a sequence of Policy elements. Let each of the $P_{\mathrm{id}}$ be a $\mathrm{policy}\left(P_{\mathrm{id}}\right)=\left\{{\mathrm{TP}}_{\mathrm{id}},\langle R_{1},\ldots R_{h}\rangle ,{\mathrm{comb}}_{p}\right\}$ where ${\mathrm{TP}}_{\mathrm{id}}$ is the Target of the policy, $\langle R_{1},\ldots R_{h}\rangle$ is the set of Rules and ${\mathrm{comb}}_{p}$ is the Combining Algorithm. Finally, let each of $R_{\mathrm{id}}$ be a $\mathrm{rule}\left(R_{\mathrm{id}}\right)=\left\{\mathrm{TR},C_{\mathrm{id}},E\right\}$ where TR is the Target of the rule, $C_{\mathrm{id}}$ is the Condition and E is the Effect.
  The set of requests RQ covers the criterion of the Policy conditions true if for each of the condition elements included in the rule R ($\mathrm{TR},C_{\mathrm{id}},E$), there exists at least an ${\mathrm{RQ}}_{i}$ that covers the condition with the true value.
- Policy conditions true and false:
  Let RQ be a set of requests $\langle {\mathrm{RQ}}_{1},\ldots {\mathrm{RQ}}_{m}\rangle$, a
  $$\mathrm{policyset}\left(\mathrm{PS}\right)=\left\{{\mathrm{comb}}_{\mathrm{ps}},\mathrm{TPS},\langle P_{1},\ldots ,P_{n}\rangle \right\}$$
  where ${\mathrm{comb}}_{ps}$ is the Combining Algorithm, TPS is the target of the policy set and $\langle P_{1},\ldots ,P_{n}\rangle$ is a sequence of Policy elements. Let each of the $P_{\mathrm{id}}$ be a $\mathrm{policy}\left(P_{\mathrm{id}}\right)=\left\{{\mathrm{TP}}_{\mathrm{id}},\langle R_{1},\ldots R_{h}\rangle ,{\mathrm{comb}}_{p}\right\}$ where ${\mathrm{TP}}_{\mathrm{id}}$ is the Target of the policy, $\langle R_{1},\ldots R_{h}\rangle$ is the set of Rules and ${\mathrm{comb}}_{p}$ is the Combining Algorithm. Finally, let each $R_{\mathrm{id}}$ be a $\mathrm{rule}\left(R_{\mathrm{id}}\right)=\left\{\mathrm{TR},C_{\mathrm{id}},E\right\}$ where TR is the Target of the rule, $C_{\mathrm{id}}$ is the Condition, and E is the Effect.
  The set of requests RQ covers the criterion of the Policy conditions true and false if for each of the condition elements included in the rule R ($\mathrm{TR},C_{\mathrm{id}},E$), there exists at least an ${\mathrm{RQ}}_{i}$ that covers the condition with the true value and at least an ${\mathrm{RQ}}_{j}$ that covers the condition with the false value.

### 7.1. Application Example of Coverage Criteria

In this section, as an example, we show the application of the coverage criteria defined in Section 7 to the policy of Listing 1 described in Section 4.3, and we provide examples of test suites able to satisfy the different proposed criteria.

#### 7.1.1. Policy Target True

Given a test suite $TS_{1}$ = {$RQ_{1}$}, where $RQ_{1}$ is the request of Listing 2, we would like to measure its coverage considering the *Policy target true* criterion. As in Listing 2, the request contains a subject having a value equal to 10. The evaluation of the request on the policy of Listing 1 provides the following results: $RQ_{1}$ covers with *True* value the Target of the PolicySet, the Target of Policy1 and the Target of the Rule1.

Indeed, because the policy-combining algorithm is FirstApplicable, i.e., it returns the result of the first applicable policy, neither Policy2 nor Rule2 are evaluated with $RQ_{1}$.

Consequently, the overall coverage measure of the *Policy targets true* criterion for $TS_{1}$ is 60%, because the policy of Listing 1 contains five targets and $RQ_{1}$ covers only three of them.

In order to increase the coverage and reach 100%, $TS_{1}$ should be enriched with a request able to reach the target of Policy2. From a practical point of view, this is possible by executing a request that makes the evaluation of the Policy1 not applicable and triggers the evaluation of Policy2. An example of such a request is provided in Listing 3, which contains a subject having a value equal to 2. Indeed, this request is able to pass the Target of the PolicySet, makes the evaluation of the Policy1 not applicable, and triggers the evaluation both of the Target of Policy2 and Rule2.

Consequently, given a test suite $TS_{2}$ = {$RQ_{1}$, $RQ_{2}$} where $RQ_{1}$ and $RQ_{2}$ are the requests of Listing 2 and Listing 3, respectively, the overall coverage measure of the *Policy targets true* criterion for $TS_{2}$ is 100% because $RQ_{2}$ is able to evaluate with a *True* value the Targets of the PolicySet, Policy2 and Rule2.

#### 7.1.2. Policy Target True and False

Given the test suite $TS_{2}$ = {$RQ_{1}$, $RQ_{2}$} where $RQ_{1}$ and $RQ_{2}$ are the requests of Listing 2 and Listing 3, respectively, we would like to measure its coverage considering the *Policy target true and false* criterion.

In this case, as already described in the previous section, $RQ_{1}$ and $RQ_{2}$ are able to evaluate with *True* value the Targets of the PolicySet, Policy1, Policy2, Rule1 and Rule2. Additionally, the request $R_{2}$ evaluates with *False* value the target of Policy1.

Consequently, the overall coverage measure of the *Policy targets true and false* criterion for $TS_{2}$ is 60%, because the policy of Listing 1 contains five targets, each one to be evaluated to *True* and *False* value, and $TS_{2}$ covers only six of them.

In order to increase the coverage and try to reach 100%, $TS_{2}$ should be enriched with additional requests. For the aim of simplicity, we report in Table 5 an example of the required requests. In particular, we considered $RQ_{3}$ having a subject value equal for instance to 16; (ii) $RQ_{4}$ with a subject value equal for instance to 11; (iii) $RQ_{5}$ with a subject value equal for instance to 8.

Consequently, given a test suite $TS_{3}$ = $TS_{2}$⋃$\left\{RQ_{3},RQ_{4},RQ_{5}\right\}$, we would like to measure its coverage considering the *Policy target true and false* criterion.

In this case

- $RQ_{3}$ is able to evaluate with *False* value the Target of the PolicySet;
- $RQ_{4}$ is able to evaluate with *False* value the Target of Policy2;
- $RQ_{5}$ is able to reach the evaluation with *True* value of the Target of Policy2 and then trigger the evaluation of the Target of the Rule2 with *False* value.

Consequently, the overall coverage measure of the *Policy targets true and false* criterion for $TS_{3}$ is 90%.

Note that it is not possible to reach 100% coverage of *Policy targets true and false* criterion, since it is not possible to have the coverage of the Target of the Rule1 with *False* value. This is because in order to trigger the evaluation of Rule1, we need a request with a subject equal to 10 that is able to evaluate with *True* value the Target of Policy1; then, this subject value is always greater than 4.

#### 7.1.3. Policy Condition True

Given the test suite $TS_{2}$ = {$RQ_{1}$, $RQ_{2}$} where $RQ_{1}$ and $RQ_{2}$ are the requests of Listing 2 and Listing 3, respectively, we would like to measure its coverage considering the *Policy condition true* criterion.

In this case, as already described in the previous section, $RQ_{1}$ and $RQ_{2}$ are able to evaluate with *True* value the Conditions of Rule1 and Rule2. Indeed, $RQ_{1}$ is able to evaluate with *True* value the condition of Rule1, whereas $RQ_{2}$ is able to evaluate with *True* value the condition of Rule2.

Consequently, the test suite $TS_{2}$ is also able to reach 100% coverage of Policy conditions true criterion.

#### 7.1.4. Policy Condition True and False

Given the test suite $TS_{4}$ = $TS_{2}$⋃$\left\{RQ_{6}\right\}$ where $RQ_{6}$ is the request of Table 5, we would like to measure its coverage considering the *Policy condition true and false* criterion.

In this case, as already described in the previous section, $RQ_{1}$, $RQ_{2}$ and $RQ_{6}$ are able to reach 75% of coverage of Policy conditions true and false criterion, since $RQ_{6}$ is able to cover with *False* value the condition of Rule2.

Note that, again, it is not possible to reach a 100% coverage measure of Policy conditions true and false criterion, since to trigger the evaluation of condition of Rule1, a subject equal to 10 is needed to evaluate to *True* value the Target of Policy1. This subject value makes always true the condition of Rule1.

## 8. Discussion and Conclusions

In this paper, we provided a formal definition of XACML syntax and semantics and defined some coverage concepts useful for verifiability and testability purposes. In particular, we revised and extended the existing definition of the abstract syntax and semantics of XACML 3.0 standard, defined a formal specification of the coverage process of the elements of the XACML policy and provided some coverage criteria useful to assess the XACML policy.

The proposed coverage criteria can have different practical implications for improving the verifiability and testability of the policy. Indeed, from a testing point of view, the policy coverage measure can describe the degree to which the policy has been exercised by a given test suite. Reaching high coverage, measured as a percentage, can increase the chance of detecting possible weaknesses or security flaws in the considered policy code. Moreover, the analysis of the policy elements that have not been covered may suggest possible improvements of the original test suite, so to increase the overall fault detection effectiveness of the test suite itself. This has been evidenced also by the application example proposed in this paper. Even if it is very simple, the application of the coverage criteria of *Target true and false* and *Condition true and false* evidenced an infeasible path in the policy specification. This does not per se represent a security flaw; however, it highlights an inaccuracy in the policy writing that should be avoided. Another practical implication of the proposed coverage criteria is the possibility of exploiting the coverage measure to reduce (or select), from a given test suite, only those test cases that have an impact on the defined coverage criteria. This is specifically important in case of regression testing because the test effort may be dedicated just to run the test cases able to maximize the coverage measure.

Finally, the proposals of this paper can also be used for the definition of test suite generation methodologies that target the 100% coverage of a specific criterion. As from the application example, the coverage of a specific target and/or condition depends on the evaluation of the previous targets and/or conditions. Thus, as a basic proposal for a test case generation algorithm able to force the execution of a specific rule, it should be considered that: it is first necessary to generate the attributes that make the Target element of the PolicySet true; successively to generate the attributes that make the Target of the current Policy true and those that make the previous Policy elements false; finally, to generate the attributes that make the Target of the Rule element true and the Targets of the previous Rules elements false.

However, many different metrics can be used to calculate policy coverage. In this paper, we provide some basic ones focusing on the evaluation of the targets and the conditions that are common critical points for most of the policies. As a future work, we would like to provide more additional specific coverage criteria as well as to perform an accurate comparison of their fault detection effectiveness so to better guide testing efforts. We are also developing ad hoc test case generation algorithms able to target the coverage criteria proposed in this paper.

## Figures and Tables

**Table 1:** SotA Advancements.

Paper (Year)	Paper Contribution	Language/ Formalism	Access Control Formalization	Coverage Metrics/ Measures	Our Advancement with Respect to SotA
[17] (2012)	The paper provides a formal semantics of XACML and its implementation based on such semantics	XACML 2.0	✓		New formalization of the XACML 3.0 standard language by considering context-free grammars as an alternative representation
[39] (2014)	The paper provides a test selection approach based on a coverage measure	XACML 2.0		✓	New coverage criteria provided and revision of the existing ones to XACML 3.0
[16] (2014)	The paper provides a formalization of the XACML3.0 using Belnap logic and D-algebra	XACML 3.0	✓		New formalization of the XACML 3.0 standard language by considering context-free grammars as an alternative representation
[27] (2015)	The paper provides a formalization of semantic differences between the combining algorithms	XACML 3.0	✓		Formalization extended to the overall structure of XACML
[28] (2015)	The paper proposes a UML profile for XACML policies specification	XACML 3.0 UML	✓		New formalization of the XACML 3.0 standard language by considering context-free grammars as an alternative representation
[42] (2018)	The paper proposes an access control infrastructure, based on a monitor engine enabling coverage criterion selection	XACML 2.0		✓	New conceived coverage criteria that could be used for improving the monitor engine. This is part of our future work
[12] (2018)	The paper defines a family of coverage criteria for XACML policies, including the MC/DC criterion	XACML 3.0		✓	Formalization of the coverage definitions considering the context-free grammars and focusing on the specific XACML 3.0 elements. Formalization of MC/DC criterion could be part of future work
[43] (2018)	The paper proposes a direct logical formalism of ABAC models using a variant of description logics and function-free first-order logic with equality	ABAC RBAC	✓		New formalization of the XACML 3.0 standard language by considering context-free grammars as an alternative representation
[29] (2020)	The paper formally models the resource attributes by dynamic description logic (DDL) and then provides means for rules conflict solving	XACML 3.0	✓		New formalization of the XACML 3.0 standard language by considering context-free grammars as an alternative representation
[31] (2021)	The paper proposes a generic AC metamodel for defining AC policies	ABAC RBAC	✓		New formalization of the XACML 3.0 standard language by considering context-free grammars as an alternative representation
[30] (2021)	The paper provides a theory for representing the semantics of rule-based policies based on the semantics of conditional expressions in their rules	ABAC RBAC	✓		New formalization of the XACML 3.0 standard language by considering context-free grammars as an alternative representation
[32] (2022)	The paper proposes an access control metamodel useful to derive various instances of AC models	ABAC RBAC	✓		New formalization of the XACML 3.0 standard language by considering context-free grammars as an alternative representation
This paper (2022)	The paper provides (i) a formalization of the XACML 3.0 standard language leveraging context-free grammars (ii) the formal definition of the coverage of the XACML 3.0 elements (iii) the definition of several coverage criteria specifically conceived for XACML 3.0 policies for testing purposes	ABAC RBAC	✓	✓	-

**Table 2:** Symbols of the grammar.

PS	PolicySet element
$T_{PS}$	Target of the PolicySet element
*P*	Policy element
PCA	PolicyCombiningAlgorithm
$T_{P}$	Target of the Policy element
$A_{\mathrm{nyOf}}$	AnyOf element
$A_{\mathrm{llOf}}$	AllOf element
*M*	Match element
$A_{\mathrm{ttrChoice}}$	Syntactic category used for deciding between $a_{d}$ and $a_{s}$
$A_{\mathrm{ttr}}$	Attribute
$S_{ubject}$	Subject’s attribute
$R_{esources}$	Resource’s attribute
$A_{ction}$	Action’s attribute
$E_{nv}$	Environment’s attribute
$A_{dHoc}$	User’s attribute
$a_{v}$	AttributeValue
$a_{d}$	AttributeDesignator
$a_{s}$	AttributeSelector
*s*	Subject’s instance
*r*	Resource’s instance
*a*	Action’s instance
*e*	Environment’s instance
$a_{h}$	Ad hoc User’s attribute instance
*R*	Rule element
$T_{R}$	Target of the Rule element
RCA	RuleCombiningAlgorithm
*C*	Condition element
${E_{X}}_{bool}$	Syntactic category $\subset E_{X}$ category for producing only expressions
	that obtain a boolean result
*A*	Apply element
$E_{X}$	Expression element
${E_{X}}_{fun}$	Syntactic category $\subset E_{X}$ category for producing only expressions
	that are functions
$F_{un}$	Function element
$E_{ffect}$	Effect that can be $p_{ermit}$ or $d_{eny}$

**Table 3:** Definition of syntactical categories with formalization on sets.

PS	=	${\left\{P\right\}}^{+}\cup \left\{\mathrm{PCA}\right\}\cup \left\{T_{\mathrm{PS}}\right\}$
*P*	=	$\left\{T_{P}\right\}\cup {\left\{R\right\}}^{+}\cup \left\{\mathrm{RCA}\right\}$
$T_{\left\{\mathrm{PS},\;P,\;R\right\}}$	=	${\left\{\mathrm{AnyOf}\right\}}^{*}$
AnyOf	=	${\left\{\mathrm{AllOf}\right\}}^{+}$
AllOf	=	${\left\{M\right\}}^{+}$
M	=	${\{\left\{a_{v}\right\}\cup \left\{el:el\in \{a_{d}\cup a_{s}\}\right\}\}}^{+}$
R	=	$\left\{E_{\mathrm{ffect}}\right\}\cup \left\{T_{R}\right\}\cup \left\{C\right\}$
$E_{X}$	=	$\left\{A,a_{v},a_{s},a_{d},F_{\mathrm{un}}\right\}$
C	=	$\{\exists !\;e\in E_{X}:e_{x\mathrm{pr}}val\left(e\right)=\left\{\begin{array}{c} T_{\mathrm{rue}}\\ F_{\mathrm{alse}} \end{array}\right.\}$
A	=	$\left\{F_{\mathrm{un}}\right\}\cup {\left\{E_{X}\right\}}^{*}$
$F_{\mathrm{un}}$	=	$\left\{F_{{\mathrm{un}}_{\mathrm{id}}}\right\}$

**Table 4:** Productions of XACML 3.0 grammar.

P={			
	$P_{S}$	→	$P_{S}\;P\;\left|\;P_{S_{1}}\;P_{S}\;\right|\;T_{PS}\;P_{CA}\;P$
	$T_{PS}$	→	$A_{\mathrm{nyOf}}\;T_{PS}\;|\;\epsilon$
	*P*	→	$R_{CA}\;T_{P}\;R\;|\;P\;R$
	$P_{CA}$	→	$p_{\mathrm{ermit}}o_{\mathrm{verrides}}\;\left|\;d_{\mathrm{eny}}o_{\mathrm{verrides}}\;\right|\;$
			$f_{\mathrm{irst}}a_{\mathrm{pplicable}}\;|$
			$o_{\mathrm{nly}}o_{\mathrm{ne}}a_{\mathrm{pplicable}}\;|\;$
			$d_{\mathrm{eny}}u_{\mathrm{nless}}p_{\mathrm{ermit}}\;|\;$
			$p_{\mathrm{ermit}}u_{\mathrm{nless}}d_{\mathrm{eny}}\;|\;$
			$o_{\mathrm{rdered}}d_{\mathrm{eny}}o_{\mathrm{verrides}}\;|\;$
			$o_{\mathrm{rdered}}p_{\mathrm{ermit}}o_{\mathrm{verrides}}$
	$T_{P}$	→	$A_{\mathrm{nyOf}}\;T_{P}\;|\;\epsilon$
	$A_{\mathrm{nyOf}}$	→	$A_{\mathrm{llOf}}\;A_{\mathrm{nyOf}}\;|\;A_{\mathrm{llOf}}$
	$A_{\mathrm{llOf}}$	→	$M\;A_{\mathrm{llOf}}\;|\;M$
	*M*	→	$F_{\mathrm{un}}\;a_{v}\;A_{\mathrm{ttrChoice}}\;M\;|\;$
			$F_{\mathrm{un}}\;a_{v}\;A_{\mathrm{ttrChoice}}$
	$A_{\mathrm{ttrChoice}}$	→	$a_{s}\;|\;a_{d}$
	$R_{CA}$	→	$p_{\mathrm{ermit}}o_{\mathrm{verrides}}\;|\;$
			$d_{\mathrm{eny}}o_{\mathrm{verrides}}\;|\;$
			$f_{\mathrm{irst}}a_{\mathrm{pplicable}}\;|$
			$d_{\mathrm{eny}}u_{\mathrm{nless}}p_{\mathrm{ermit}}\;|\;$
			$p_{\mathrm{ermit}}u_{\mathrm{nless}}d_{\mathrm{eny}}\;|\;$
			$o_{\mathrm{rdered}}d_{\mathrm{eny}}o_{\mathrm{verrides}}\;|\;$
			$\;o_{\mathrm{rdered}}p_{\mathrm{ermit}}o_{\mathrm{verrides}}$
	*R*	→	$E_{\mathrm{ffect}}\;T_{R}\;C$
	$T_{R}$	→	$T_{R}\;A_{\mathrm{nyOf}}\;|\;\epsilon$
	$E_{X}$	→	$A\;|\;a_{s}\;|\;a_{v}\;\left|\;a_{d}\;\right|\;F_{\mathrm{un}}$
	${E_{X}}_{\mathrm{fun}}$	→	$F_{\mathrm{un}}$
	$F_{\mathrm{un}}$	→	$s_{\mathrm{tring}}e_{\mathrm{qual}}\;\left|\;s_{\mathrm{tring}}g_{\mathrm{reater}}t_{\mathrm{han}}\;\right|\;$
			$s_{\mathrm{tring}}l_{\mathrm{ess}}t_{\mathrm{han}}\;|$
			$s_{\mathrm{tring}}g_{\mathrm{reater}}t_{\mathrm{han}}o_{r}e_{\mathrm{qual}}\;|\;$
			$s_{\mathrm{tring}}l_{\mathrm{ess}}t_{\mathrm{han}}o_{r}e_{\mathrm{qual}}$
			$i_{\mathrm{nteger}}e_{\mathrm{qual}}\;\left|\;i_{\mathrm{nteger}}g_{\mathrm{reater}}t_{\mathrm{han}}\;\right|$
			$i_{\mathrm{nteger}}l_{\mathrm{ess}}t_{\mathrm{han}}\;|$
			$i_{\mathrm{nteger}}g_{\mathrm{reater}}t_{\mathrm{han}}o_{r}e_{\mathrm{qual}}\;|\;$
			$i_{\mathrm{integer}}l_{\mathrm{ess}}t_{\mathrm{han}}o_{r}e_{\mathrm{qual}}$
	$E_{\mathrm{ffect}}$	→	$p_{\mathrm{ermit}}\;|\;d_{\mathrm{eny}}$
}			

**Table 5:** Subject value for each request.

Request	*RQ*_1_	*RQ*_2_	*RQ*_3_	*RQ*_4_	*RQ*_5_	*RQ*_6_
Subject Value	10	2	16	11	8	4

## Data Availability

Data sharing not applicable.
